# Improving Capacity and Consent to Treatment Recording, Park House Hospital

**DOI:** 10.1192/bjo.2022.465

**Published:** 2022-06-20

**Authors:** Rebecca McKnight, Rachel Fu

**Affiliations:** Greater Manchester Mental Health NHS Foundation Trust, Manchester, United Kingdom

## Abstract

**Aims:**

Re-audit for adherence of all inpatient wards at Park House Hospital to Trust Consent to Treatment policy. Improve hospital compliance to Trust Consent to Treatment policy. Reduce prescribing errors. Improve trainee confidence and knowledge of Consent to Treatment

**Methods:**

- Cross sectional audit.
- Data collected between 8th and 12th November 2021
- All wards in Park House Hospital
- 5 patient records and medication charts reviewed per ward.
- Proforma used.
- Data analysed using Excel.
- Interactive teaching on Consent to Treatment delivered by Dr McKnight to Core Psychiatry Trainees on 3rd July 2020.
- Dr McKnight presented the original audit data and consulted the Pharmacists and Consultants to assess and improve ward systems for recording Consent to Treatment. (26th May and 30th April 2021).
- Dr McKnight presented to Greater Manchester Mental Health, Mental Health Act and Mental Capacity Act Quality Improvement Group (30th June 2020).

**Results:**

- No wards had 100% capacity forms documented, kept in medication charts and uploaded to Paris.
- 7/9 wards had 100% compliance for completing T2/3/S62 forms.
- 6/9 wards had 100% compliance rate for retaining the T2/3/S62 forms in the medication charts.
- 78% T2/3/S62 forms were uploaded to PARIS.
- 80% medication charts matched T2/3 forms.

When Dr McKnight asked trainees, “Do you feel confident with your knowledge of consent to treatment” only 24% answered yes, 35% answered no and 41% a little.

When asked, “Do you check Consent to treatment forms before prescribing?” 32% answered yes, 24% no, 34% sometimes and 10% that they didn't know what they were.

During the post-teaching quiz, trainees were asked, “Has this teaching session improved your knowledge and confidence regarding Consent to Treatment?” 91% answered yes, 0% answered no and 9% answered a little.

Discussion with Consultants and Pharmacists concluded that it may be beneficial for wards to include Capacity to Consent and Consent to Treatment within ward round proformas

**Conclusion:**

- The two main concerns of the initial audit and re-audit, relate to Treatment Capacity and Consent forms compliance and prescribing.
- New trainees rotate into the Trust every 6 months and levels of knowledge surrounding Consent to Treatment varies depending on trainee experience. Trainees require teaching on Consent to Treatment as part of their induction and teaching programme.
- Based on the multidisciplinary nature of ensuring compliance to Consent to Treatment the authors propose monthly ward auditing of Consent to Treatment, which they believe will lead to better compliance rates across the hospital.

